# Dual inhibition of complement component 5 and leukotriene B4 by topical rVA576 in atopic keratoconjunctivis: TRACKER phase 1 clinical trial results

**DOI:** 10.1186/s13023-021-01890-6

**Published:** 2021-06-11

**Authors:** Sara Sánchez-Tabernero, Julia Fajardo-Sanchez, Wynne Weston-Davies, Mohit Parekh, Jaime Kriman, Stephen Kaye, Sajjad Ahmad

**Affiliations:** 1 Moorfields Eye Hospital, 51 North Block, 5 Chicheley Street, London, SE1 7PJ UK; 2 Akari Therapeutics PLC, London, UK; 3 UCL Institute of Ophthalmology, London, UK; 4 St Paul’s Eye Unit, Royal Liverpool Hospitals, Liverpool, UK

**Keywords:** Allergic eye disease, Atopic keratoconjunctivitis, Complement, C5, Leukotriene, Nomacopan, Clinical trial

## Abstract

**Purpose:**

To evaluate the safety and preliminary efficacy of topical rVA576, a dual inhibitor of complement component 5 (C5) and leukotriene B4 (LTB4), in patients with recalcitrant atopic keratoconjunctivitis (AKC) in the open label phase 1 TRACKER clinical trial.

**Methods:**

Three patients diagnosed with moderate or severe AKC who had been on maximal topical treatment (antihistamines and ciclosporin) for at least three months prior to entry, and showed persistent symptoms and signs of inflammation, were recruited into the trial. Patients received rVA576 eye drops twice a day for 8 weeks. Patients were seen at baseline and weeks 1, 2, 4, 6 and 8. Safety data was recorded and a composite sum score of symptoms and signs was obtained. This score comprised symptoms such as itching, mucous discharge and photophobia, and conjunctival and corneal signs such as hyperemia, tarsal papillae, punctate keratitis and corneal neovascularization, all rated individually from 0 to 3 for a maximum score of 33.

**Results:**

Two of the three patients completed the initial open label phase of the trial. The third patient was unable to attend appointments and terminated the study early at day 14. Topical rVA576 was well tolerated with no serious adverse events reported. There was an average improvement in overall clinical score of 53%, composed of an improvement in symptoms of 65% [63.64–66.67%] and signs of 40% [40–40.12%] by day 56.

**Conclusions:**

In this open label phase 1 TRACKER trial, rVA576 eye drops were well tolerated and showed a response across signs and symptoms of active inflammation. This study is exploratory but supports topical rVA576 safety and shows promising efficacy for recalcitrant AKC. A phase 2 randomised control trial is currently underway.

**Supplementary Information:**

The online version contains supplementary material available at 10.1186/s13023-021-01890-6.

## Introduction

The recombinant protein rVA576 (commercially known as Nomacopan; Akari Therapeutics, New York, USA) is derived from a protein discovered in the saliva of the Ornithodoros moubata tick, also known as OMCI [1], where it counteracts the host inflammatory response and allows the parasite to obtain repeated blood meals. For this reason, the molecule needs to be well-tolerated and needs to maintain its inhibitory effect in the host repeatedly exposed to the same molecule. Nomacopan binds to the complement C5 molecule, thereby interfering with the interaction between C5 and the C5 convertases and thus preventing its cleavage to C5a and C5b. This generates a total blockade of the terminal complement system and prevents the formation of the membrane attack complex (MAC) [1]. Nomacopan has a second independent action: it binds and inactivates leukotriene B4 (LTB4), a potent white blood cell chemotactant, at a separate, internalised binding site. This is a high-affinity binding that outcompetes the natural receptors, BLT1 and BLT2 and results in an effectively irreversibly bound complex. Both C5 and leukotriene B4 have been implicated in ocular surface inflammation [2, 3].

Atopic keratoconjunctivitis (AKC) is a severe chronic allergic eye disease that primarily affects the adult population. Compared to other forms of allergy, AKC is characterised by a more chronic and insidious course, with recurrent episodes of inflammation that can lead to corneal scarring and neovascularisation, and ultimately vision loss. AKC involves mast cell activation secondary to the predominance of inflammatory mediators such as eosinophils and Th2-type cytokines [4]. Management consists of a stepladder approach of topical treatment in the first instance, including lubricants, antihistamines, immunomodulators (e.g. ciclosporin A) and intermittent short-term courses of topical steroids. However, systemic immunosuppression can become necessary in unresponsive patients or those requiring recurrent or long-term topical steroid treatment. There is a requirement for alternative topical anti-inflammatory agents as an intermediate or alternative treatment step prior to systemic therapy. This open label study presents safety and efficacy data of rVA576 as a novel topical treatment for AKC which fills this therapeutic space in the stepladder of management.

## Methods

### Inclusion/exclusion criteria and follow up

This study was an open label phase 1 clinical trial of topical rVA576 eye drops in patients with moderate to severe AKC with a treatment period of 8 weeks and a further follow-up of 4 weeks beyond rVA576 therapy. It is to be followed up by a randomized, double blinded, placebo-controlled phase 2 clinical trial (ongoing at present).

Patients included were aged 18 and above who had been on maximal topical therapy for at least three months without improvement but were not receiving systemic immunotherapy. A full list of inclusion and exclusion criteria has been provided in Table 1. Topical rVA576 2.5 mg/mL eye drops were administered to both eyes twice daily for 56 days (8 weeks) with a further follow-up of 4 weeks after rVA576 treatment cessation. Patients continued on the topical medication they were using before entering the trial. Rescue medication in the form of topical steroids was allowable if considered clinically necessary and was documented.

**Table 1:** Inclusion and exclusion criteria

Inclusion criteria	Exclusion criteria
1. Aged 18 and above	1. Eye surface disease other than AKC
2. Diagnosis of moderate to severe AKC with a composite symptom/sign score from one eye of ≥ 18 out of 33 (see Clinical Scoring 17.1)	2. Contact lens use during the study
3. Will have had maximal topical therapy for at least 3 months without improvement but will not currently be receiving systemic immunotherapy	3. Complete or partial tarsorrhaphy. If such a procedure becomes necessary during the course of the trial patients may remain in the trial providing that at least 50% of the eye surface remains visible to slit lamp examination
4. History of atopy other than ocular (dermatitis, asthma, hay fever)	4. Ankyloblepharon of any degree at entry to the trial
5. Willing to give informed consent	5. Known or suspected ocular malignancy
6. Willing to use highly effective contraceptive precautions for the duration of the study and for 90 days after the last dose of IMP	6. Active ocular infection at entry to the trial. Patients with eye surface bacterial, viral, fungal or protozoal infection may enter the trial after elimination of the infection as confirmed by eye swabs
7. Willing to avoid prohibited medications for duration of study (see list of prohibited medications)	7. Known or suspected uveitis
8. All patients in the study must be receiving maximum topical ciclosporin (Ikervis)	8. Participation in any other clinical trial within 1 month of enrolment
9. All patients will be receiving a topical antihistamine (olopatadine hydrochloride) twice daily	9. Use of any of the following prohibited medications: Eculizumab Any other investigational complement inhibitor whether systemic or topical (e.g. RA101495) Montelukast Zafirlukast Pranlukast Zileuton Hypericum perforatum (St John’s wort)
10. All patients may use an eye lubricant pro re nata (p.r.n.)	10. Corneal perforation
	11. Uncontrolled glaucoma (increase in dose of glaucoma medication or surgical intervention for glaucoma within 3 months prior to entry)
	12. Pregnancy (females)
	13. Breast feeding (females)
	14. Known allergy to ticks or severe reaction to arthropod venom (e.g. bee or wasp venom)
	15. Use of topical optical steroids within 14 days of the Screening Visit
	16. Failure to satisfy the PI of suitability to participate for any other reason

### Data acquisition and analysis

The primary trial objective was to demonstrate the safety and tolerability of rVA576 when given by topical ocular administration to patients with AKC. Tolerability of the study drug was assessed by patient diary cards. The diary cards measured the comfort profile by asking the patient to describe in one word how comfortable or not they felt immediately after the instillation of the study drug and after 1, 2, 3 and 5 min. Safety data was assessed by review of adverse event reporting.

Safety data was recorded and a composite sum score of signs and symptoms was obtained. This scoring system is described in Table 2, adapted from Akpek et al. [5]. The data comprises symptoms such as itching, mucous discharge and photophobia, and conjunctival and corneal signs such as hyperemia, tarsal papillae, punctate keratitis and corneal neovascularization, rated individually from 0 to 3 for a maximum score of 33. The score was recorded at baseline, at follow-up visits and at the end of the treatment period.

**Table 2:** Symptoms and signs scoring system, adapted from Akpek et al. [5]

Symtoms	0	1	2	3
Itch	No desire to rub or scratch the eye	Occasional desire to rub or scratch	Frequent need to scratch or rub the eye	Constant need to rub or scratch the eye
Tearing	Normal tear production	Positive sensation of fullness of the conjunctival sac without tears spilling over the lid margin	Intermittent, infrequent spilling of tears over the lid margin	Constant, or nearly constant, spilling of tears over the lid margins
Discomfort (including burning, stinging, and foreign body sensations)	Absent	Mild	Moderate	Severe
Discharge	No abnormal discharge	Small amount of mucoid discharge noted in the lower cul-de-sac	Moderate amount of mucoid discharge noted in the lower cul-de-sac and in the marginal tear strip; presence of crust upon awakening	Eyelids tightly matted together upon awakening, requiring warm soaks to pry lids apart; warm soaks necessary to clean eyelids during the day
Photophobia	No difficulty experienced	Mild difficulty with light causing squinting	Moderate difficulty, necessitating dark glasses	Extreme photophobia, causing the patient to stay indoors; cannot stand natural light even with dark glasses
Signs				
Bulbar conjunctival hyperemia	Absent	Mild	Moderate	Severe
Tarsal conjunctival papillary hypertrophy	No evidence of papillary formation	Mild papillary hyperemia	Moderate papillary hypertrophy with edema of the palpebral conjunctiva and hazy view of the deep tarsal vessel	Severe papillary hypertrophy obscuring the visualization of the deep tarsal vessels
Punctate keratitis (superficial epithelial keratitis and punctate staining of the cornea with fluorescein)	No evidence of punctate keratitis	One quadrant of punctate keratitis	Two quadrants of punctate keratitis	Three or more quadrants of punctate keratitis
Neovascularization of cornea (new vessel formation, crossing the limbus onto the clear cornea by 2 mm)	No evidence of new vessel formation	Presence of neovascularization in 1 quadrant of cornea	Presence of neovascularization in 2 quadrants of cornea	Presence of neovascularization in 3 quadrants of cornea
Cicatrizing conjunctivitis (superficial scarring of the conjunctiva)	No evidence of cicatrization	Presence of subepithelial fibrosis	Presence of fornix foreshortening	Symblepharon formation
Blepharitis (hyperemia and edema of eyelid skin with meibomian gland dysfunction)	No evidence of blepharitis	Presence of mild redness and edema of the eyelid with meibomian gland dysfunction	Moderate inflammation with hyperemia, scales, and scurf of eyelid skin and toothpaste phenomenon	Severe inflammation, with cracks in the eyelid skin, loss of eyelashes, and lid oedema

The secondary objective was to evaluate efficacy. Efficacy was assessed in the eye judged to be worst affected by the patient, or if equally affected, in the right eye.

## Results

Three patients were enrolled in the study. Two of them completed 56 days (8 weeks) of treatment and one completed 14 days and then withdrew for reasons unrelated to the study treatment. The two patients who completed the study continued a further 4-week follow-up after cessation of rVA576 use. The drops were found to be comfortable and well-tolerated throughout the trial for all three patients. Post-instillation comfort was reported by patients as excellent with high levels of acceptance of eye drops, which were described as comfortable. Tolerability in ocular surface inflammatory disease is important to ensure long-term compliance of therapy. There were no serious adverse events (SAE) reported in this trial.

**Fig. 1:**
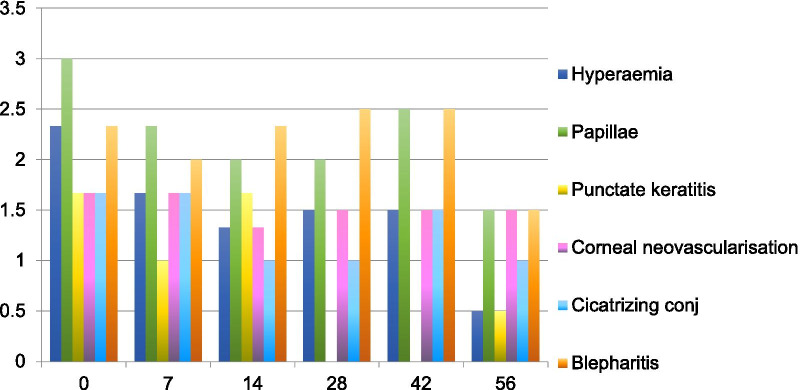
Mean sign scores over time

**Fig. 2:**
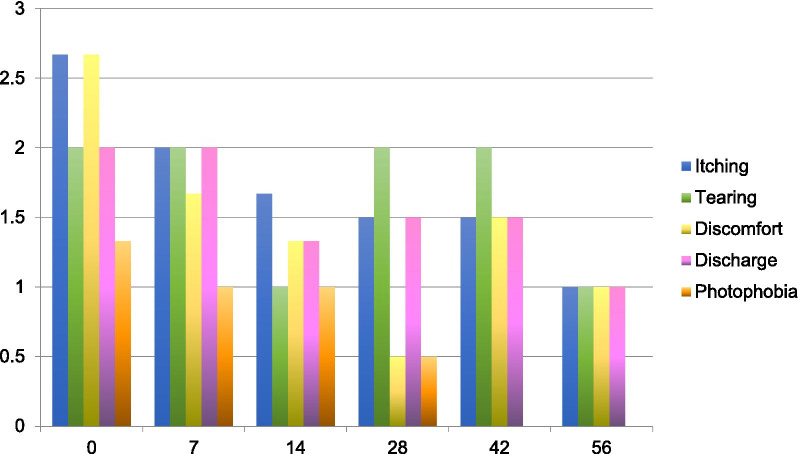
Mean symptom scores over time

**Fig. 3:**
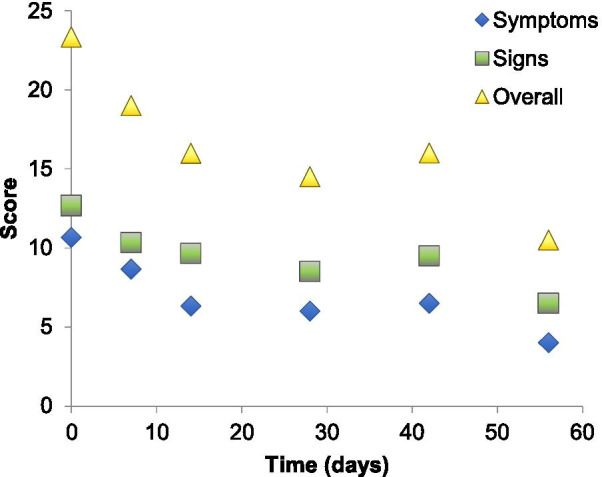
Average symptom, sign and overall scores over time. There was an average improvement in overall clinical score of 53%, composed of an improvement in symptoms of 65% [63.64–66.67%] and signs of 40% [40–40.12%] by day 56

All patients saw a reduction in average sign and symptom scores by day seven of treatment (Figs. 1, 2, Additional file 1: Table S3 for complete scores). Improvement from baseline was maintained until the end of the treatment period (day 56) and persisted after discontinuation of treatment (day 84). There was an overall improvement in clinical score of 53% composed of an improvement in symptoms of 65% [63.64–66.67%] and signs of 40% [40–40.12%] by Day 56 (Fig. 3). By day 84 the average clinical score improvement was of 45%, composed of an improvement in symptoms of 71% and signs of 29%, again compared to baseline scores. Patient 1 received a rescue course of topical steroids (unpreserved dexamethasone 0.1% four times a day for one week, then three times a day for one week, then twice a day for one week, then once a day for one week) to both eyes at day 50 because of a flare-up of his AKC symptoms.

## Discussion

TRACKER (Topical rVA576 for TReatment of Atopic KERatoconjunctivitis) is the first clinical trial to evaluate rVA576 as a treatment for any ocular disease (first-in-eye study). It was administered topically to patients with recalcitrant atopic keratoconjunctivitis. Systemic rVA576 has already been studied for the treatment of non-ocular disease. Paroxysmal nocturnal haemoglobinuria (PNH) is one such disease where subcutaneous injections of rVA576 have been used with success [6]. PNH is a life threatening disease that results in complement-induced haemolysis. Treatment with rVA576 achieved significant reduction of haemolytic complement to less than 8 U Eq/ml (the lower limit of quantification) while symptoms and laboratory markers of haemolysis improved. A phase IIa open label single arm study of rVA576 in adult mild to moderate bullous pemphigoid has just been completed.

The data from this study suggests rVA576 is safe and well tolerated as an eye drop medication. In terms of efficacy, albeit in a small number of patients, the results of this first-in-eye open label clinical trial of topical rVA576 showed clinically relevant reduction across signs and symptoms of active inflammation in a group of patients with recalcitrant disease. It is therefore a promising new topical treatment for atopic keratoconjunctivitis filling a stepladder space of clinical need. Limitations of this trial include a small number of participants and lack of placebo comparison. However, part 2 of the TRACKER clinical trial is underway with a control arm to the study.

## Conclusion

Overall the results of this first-in-eye open label trial are novel and important, showing a potentially relevant role for rVA576 eye drops in the treatment of atopic keratoconjunctivitis. This work also highlights the need for further investigation of the role of complement in inflammatory ocular surface disease.

## Supplementary Information

**Additional file 1.** Supplementary table with complete patient scores.

## Data Availability

The data that support the findings of this study are available from Akari Therapeutics but restrictions apply to the availability of these data, which were used under license for the current study, and so are not publicly available. Data are however available from the authors upon reasonable request and with permission of Akari Therapeutics.

## References

[1] Nunn MA, Sharma A, Paesen GC, Adamson S, Lissina O, Willis AC, Nuttall PA (2005). Complement inhibitor of C5 activation from the soft tick *Ornithodoros moubata*. J Immunol.

[2] Mondino BJ, Sumner HL (1990). Generation of complement-derived anaphylatoxins in normal human donor corneas. Investig Ophthalmol Vis Sci.

[3] Nathan H, Naveh N, Meyer E (1994). Levels of prostaglandin E2 and leukotriene B4 in tears of vernal conjunctivitis patients during a therapeutic trial with indomethacin. Doc Ophthalmol.

[4] Mishra GP, Tamboli V, Jwala J, Mitra AK (2011). Recent patents and emerging therapeutics in the treatment of allergic conjunctivitis. Recent Pat Inflamm Allergy Drug Discov.

[5] Akpek EK, Dart JK, Watson S, Christen W, Dursun D, Yoo S, O'Brien TP, Schein OD, Gottsch JD (2004). A randomized trial of topical cyclosporin 0.05% in topical steroid-resistant atopic keratoconjunctivitis. Ophthalmology.

[6] Schols S, Nunn MA, Mackie I, Weston-Davies W, Nishimura JI, Kanakura Y, Blijlevens N, Muus P, Langemeijer S (2019). Successful treatment of a PNH patient non-responsive to eculizumab with the novel complement C5 inhibitor coversin (nomacopan). Br J Haematol.

[7] Jore MM, Johnson S, Sheppard D, Barber NM, Li YI, Nunn MA, Elmlund H, Lea SM (2016). Structural basis for therapeutic inhibition of complement C5. Nat Struct Mol Biol.

[8] Fredslund F, Laursen NS, Roversi P, Jenner L, Oliveira CL, Pedersen JS, Nunn MA, Lea SM, Discipio R, Sottrup-Jensen L, Andersen GR (2008). Structure of and influence of a tick complement inhibitor on human complement component 5. Nat Immunol.

