# Supplementary material for: Dual inhibition of complement component 5 and leukotriene B4 by topical rVA576 in atopic keratoconjunctivis: TRACKER phase 1 clinical trial results
Source: Orphanet J Rare Dis. 2021 Jun 11;16:270. doi: 10.1186/s13023-021-01890-6 (PMC8196439; doi:10.1186/s13023-021-01890-6)
Supplement: Supplementary file 1 — Additional file 1. Supplementary table with complete patient scores. [file 13023_2021_1890_MOESM1_ESM.docx]

Supplementary Table 3: Patient scores

|  |  | **PATIENT 1** | |  |  |  |  |  |
| --- | --- | --- | --- | --- | --- | --- | --- | --- |
| SYMPTOMS | | Baseline | Day 7 | Day 14 | Day 28 | Day 42 | Day 56 |  |
|  | ITCH | 3 | 2 | 2 | 2 | 2 | 1 |  |
|  | TEARING | 3 | 2 | 1 | 2 | 3 | 1 |  |
|  | DISCOMFORT | 3 | 1 | 0 | 0 | 3 | 1 |  |
|  | DISCHARGE | 2 | 1 | 0 | 2 | 2 | 1 |  |
|  | PHOTOPHOBIA | 0 | 0 | 0 | 0 | 0 | 0 |  |
| SIGNS | BULBAR CONJ. HYPERAEMIA | 3 | 2 | 2 | 2 | 2 | 1 |  |
|  | TARSAL CONJ. PAPILLARY HYPERTROPHY | 3 | 3 | 2 | 3 | 3 | 2 |  |
|  | PUNCTATE KERATITIS | 0 | 0 | 1 | 0 | 0 | 0 |  |
|  | NEOVASC. OF CORNEA | 1 | 1 | 1 | 1 | 1 | 1 |  |
|  | CICATRIZING CONJ. | 2 | 2 | 2 | 1 | 2 | 1 |  |
|  | BLEPHARITIS | 3 | 2 | 2 | 2 | 3 | 2 |  |
| TOTAL |  | 23 | 16 | 13 | 15 | 21 | 11 |  |
|  |  |  |  |  |  |  |  |  |
|  |  | **PATIENT 2** | |  |  |  |  |  |
| SYMPTOMS |  | Baseline | Day 7 | Day 14 | Day 28 | Day 42 | Day 56 |  |
|  | ITCH | 2 | 1 | 0 | 1 | 1 | 1 |  |
|  | TEARING | 3 | 2 | 1 | 2 | 1 | 1 |  |
|  | DISCOMFORT | 2 | 1 | 1 | 1 | 0 | 1 |  |
|  | DISCHARGE | 3 | 2 | 2 | 1 | 1 | 1 |  |
|  | PHOTOPHOBIA | 2 | 1 | 1 | 1 | 0 | 0 |  |
| SIGNS | BULBAR CONJ. HYPERAEMIA | 1 | 0 | 0 | 1 | 1 | 0 |  |
|  | TARSAL CONJ. PAPILLARY HYPERTROPHY | 3 | 1 | 3 | 1 | 2 | 1 |  |
|  | PUNCTATE KERATITIS | 2 | 0 | 1 | 0 | 0 | 1 |  |
|  | NEOVASC. OF CORNEA | 1 | 1 | 1 | 2 | 2 | 2 |  |
|  | CICATRIZING CONJ. | 1 | 1 | 1 | 1 | 1 | 1 |  |
|  | BLEPHARITIS | 2 | 2 | 2 | 3 | 2 | 1 |  |
| TOTAL |  | 22 | 12 | 13 | 14 | 11 | 10 |  |
|  |  |  |  |  |  |  |  |  |

|  |  | **PATIENT 3** | |  |  |  |  |  |
| --- | --- | --- | --- | --- | --- | --- | --- | --- |
|  |  | Baseline | Day 7 | Day 14 | Day 28 | Day 42 | Day 56 |  |
| SYMPTOMS | ITCH | 3 | 3 | 3 |  |  |  |  |
|  | TEARING | 0 | 2 | 1 |  |  |  |  |
|  | DISCOMFORT | 3 | 3 | 3 |  |  |  |  |
|  | DISCHARGE | 1 | 3 | 2 |  |  |  |  |
|  | PHOTOPHOBIA | 2 | 2 | 2 |  |  |  |  |
| SIGNS | BULBAR CONJ. HYPERAEMIA | 3 | 3 | 2 |  |  |  |  |
|  | TARSAL CONJ. PAPILLARY HYPERTROPHY | 3 | 3 | 1 |  |  |  |  |
|  | PUNCTATE KERATITIS | 3 | 3 | 3 |  |  |  |  |
|  | NEOVASC. OF CORNEA | 3 | 3 | 2 |  |  |  |  |
|  | CICATRIZING CONJ. | 2 | 2 | 0 |  |  |  |  |
|  | BLEPHARITIS | 2 | 2 | 3 |  |  |  |  |
| TOTAL |  | 25 | 29 | 22 |  |  |  |  |
|  |  |  |  |  |  |  |  |  |
|  |  |  |  |  |  |  |  |  |
